# The trinity of the cortical actin in the initiation of HIV-1 infection

**DOI:** 10.1186/1742-4690-9-45

**Published:** 2012-05-28

**Authors:** Mark Spear, Jia Guo, Yuntao Wu

**Affiliations:** 1 National Center for Biodefense and Infectious Diseases, Department of Molecular and Microbiology, George Mason University, Manassas, VA, 20110, USA

**Keywords:** HIV-1, gp120, Nef, Actin, Cofilin, LIMK1, Arp2/3, CD4, CXCR4, Chemokine, Chemotaxis

## Abstract

For an infecting viral pathogen, the actin cortex inside the host cell is the first line of intracellular components that it encounters. Viruses devise various strategies to actively engage or circumvent the actin structure. In this regard, the human immunodeficiency virus-1 (HIV-1) exemplifies command of cellular processes to take control of actin dynamics for the initiation of infection. It has becomes increasingly evident that cortical actin presents itself both as a barrier to viral intracellular migration and as a necessary cofactor that the virus must actively engage, particularly, in the infection of resting CD4 blood T cells, the primary targets of HIV-1. The coercion of this most fundamental cellular component permits infection by facilitating entry, reverse transcription, and nuclear migration, three essential processes for the establishment of viral infection and latency in blood T cells. It is the purpose of this review to examine, in detail, the manifestation of viral dependence on the actin cytoskeleton, and present a model of how HIV utilizes actin dynamics to initiate infection.

## Review

### Background

The cytoskeleton is a dynamic structure composed of microfilaments (filamentous actin or F-actin), intermediate filaments, and microtubules that are mainly responsible for defining cell shape, mediating motility, and transporting macromolecules and organelles. The actin cytoskeleton, in particular, has been shown to be the driving force for cell motility and migration [1], and is involved in multiple cellular processes in the host immune response [2,3]. As an integral component of intracellular molecular networks, the actin cytoskeleton is also a target for pathogens, as originally recognized in studies of the human pathogens *Listeria monocytogenes*[2,3] and vaccinia virus [4]. Complex viruses such as vaccinia virus (200 kb dsDNA genome, encoding approximately 250 genes) or baculovirus (130 kb dsDNA genome, encoding approximately 150 genes) utilize dedicated viral proteins such as A36R and P78/83 to hijack the cellular actin polymerization process to propel essential viral activities [4,5]. In vaccina viral infection, the virus mimics normal cellular signaling pathways to mediate actin-based motility for spreading between cells. This is achieved through tyrosine phosphorylation of A36R that recruits host N-Wiskott-Aldrich syndrome protein (N-WASP) to the site for actin assembly [4]. In baculovirus, actin-based viral motility is required for viral intracellular migration and nuclear entry early in the infection process and viral spread towards the end of the viral life cycle [6]. Remarkably, to trigger actin polymerization in the nucleus, the virus translocates the host Arp2/3 complex into the nucleus where it is activated by P78/83, a viral mimic of the host WASP protein [5]. However, small viruses such as HIV (9 kb ssRNA genome, encoding 9 genes) may not have proteins with such a high specificity for the actin network. It remains unknown if any of the HIV-encoded essential or accessory proteins has a dedicated role for utilizing the actin network.

From a historical perspective, early indications that HIV may interact with cytoskeletal elements came from several studies. Hottiger *et al.* described a potential interaction between actin and either the large subunit of HIV-1 reverse transcriptase or Pol polyprotein precursor [7]. Rey *et al.* described cofractionation and interaction of HIV Gag with F-actin [8], which was later mapped to the Gag nucleocapsid (NC) domain [9-11]. HIV integrase and Nef were also found to bind to actin [12-14]. However, these and similar findings were not given sufficient attention for several reasons: the degree to which these interactions were specific was uncertain, as actin can interact non-specifically with many proteins; secondly, the lack of a clear mechanistic process by which actin could contribute to HIV infection may have lessened overall interest in the role of the cytoskeleton; and, last but not least, targeting actin as an antiretroviral strategy is typically viewed as problematic since targeting fundamental cellular elements would result in severe cytotoxicity. As such, the therapeutic applicability of these findings was questionable.

On the other hand, there were some indications that interacting with the actin cytoskeleton may not be so critical for the virus. It has been known that pseudotyping HIV particles with the vesicular stomatitis virus glycoprotein (VSV-G) can generate highly infectious particles in the laboratory [15]. VSV-G pseudotyping mediates viral entry through endocytosis, circumventing the viral receptors and the actin cortex. These results suggest that for HIV, interacting with the cortical actin may be unnecessary and avoidable, as least at postentry steps in transformed cells. However, two pieces of evidence suggest that these observations may not reflect the genuine cellular environment that the virus encounters *in vivo*. Firstly, the HIV Nef protein, a critical factor involved in HIV pathogenesis, no longer plays a role during VSV-G-pseudotyped virus infection of target cells [15-17]. Coincidentally, it was found that treatment of cells with actin inhibitors also eliminated Nef-mediated enhancement of viral infectivity [18]. These results suggest that at least one viral protein, Nef, may need the involvement of actin to facilitate viral infection and pathogenesis. Secondly, it was recently demonstrated by two independent laboratories that the VSV-G-pseudotyped virus, although highly infectious for transformed cells, is not capable of infecting resting CD4 T cells, the primary targets of HIV infection [19,20]. The VSV-G-pseudotyped virus was either incapable of entering resting CD4 T cells [20] or was destroyed in the endocytic pathway within 1–2 days following entry [19]. If the endocytic pathway [21] is defective in resting T cells [19,20], HIV envelope-mediated membrane fusion would directly deliver the viral core in front of the cortical actin [22-26]; an encounter and engagement with the cortical actin is unavoidable, either actively or passively.

Cortical actin dynamics in T cells are normally controlled by chemokine receptor binding and signal transduction [1]. The fact that HIV selects two chemokine receptors, CCR5 and CXCR4, as the main entry coreceptors implies an urgent need for the virus to engage the chemotactic process to initiate infection [19]. This has been explicitly demonstrated recently in HIV-1 latent infection of resting CD4 T cells [27,28]. In this process, HIV utilizes gp120 to trigger the activation of actin regulators such as cofilin and the LIM domain kinase 1 (LIMK1) to increase actin dynamics [27,28]. It has become evident that a dynamic cytoskeleton is important for viral entry, postentry DNA synthesis, and nuclear migration in resting T cells [19,27-30]. These recent studies have rekindled an interest in the role of the actin cytoskeleton in HIV biology and in viral pathogenesis [31-35]. It is the purpose of this review to integrate recent findings with the scattered pieces of data from the past, and to provide a prototype model to facilitate our understanding of this complicated process. In addition, the pathogenic implications of HIV hijacking host actin activity in blood CD4 T cells will also be discussed in this review.

### Role of actin in HIV-1 entry

HIV entry into target cells requires serial engagement of the primary viral receptor, CD4 [36-41], and coreceptor, CXCR4 [42] or CCR5 [43-48]. This binding event ultimately culminates in viral fusion, wherein the viral core is deposited into the cytoplasmic compartment. A debatable question is to what extent cellular factors determine the outcome of viral receptor/coreceptor engagement. Lapham *et al.*[49] and others [50-52] produced some of the first data indicating that CD4/coreceptor colocalization at the cell surface is an active process. Specifically, the association of CD4 and the coreceptors is increased upon gp120 binding in T cells or macrophages. It was also speculated that this association might occur in membrane microdomains [49]. Ugolini *et al.* observed that CD4/CXCR4 colocalization was increased in response to HIV-1 soluble gp120 treatment [53]. However, this response to gp120 treatment was not experimentally correlated with any infection process, and, as such, its contribution to HIV entry is rather speculative. In addition to receptor colocalization, there is growing evidence indicating that viral entry may be dependent on the actin cytoskeleton as well as dynamic inputs associated with CD4 and coreceptor signal transduction [27,28,53-59]. However, the modeling of an entry requirement for active receptor clustering and receptor signaling must take into account existing evidence on the constitutive association and juxtaposition of CD4 with CCR5 or CXCR4 in the absence of viral binding [49,53,60-63]. Xiao *et al.* described a constitutive association between CD4 and CCR5 or CXCR4 in cell lines, primary T cells and macrophages [61], but did not observe a significant increase in CD4/CCR5 coimmunoprecipitation upon gp120 binding [61]. This result was corroborated by a fluorescent resonance energy transfer (FRET) study by Toth *et al.*, showing that CD4 and CXCR4 are constitutively associated to a significant extent on the cell surface, and this interaction is not altered by gp120 binding [64]. Similarly, Singer *et al.* observed a constitutive association between CD4 and CXCR4 or CCR5 on microvilli in PBMC-derived macrophages and T cells [62]. Immuno-gold electron microscopy showed that CD4 and coreceptors exist as homogenous microclusters that are typically within 100 nm, or one viral diameter, of each other [62]. These findings were again supported by a Fluorescence Recovery After Photo Bleaching (FRAP) study performed by Baker *et al.*, who described a constitutive association of CD4 with multiple CCR5 at the plasma membrane [63]. From these studies, a question arises as to why HIV requires an active process to cocap receptors when these molecules are already abundantly associated. An explanation given by Baker *et al.* is that although the basal CD4-CCR5 interaction is maximal, the addition of gp120 brings the two receptors closer [63]. This process may be energy-dependent and require active participation of other cellular components [65]. Indeed, it has been suggested that an active infection process requires clustering of multiple gp120 with multiple CD4 and coreceptors [61,66-69]. The recruitment of the gp120-CD4-coreceptor complex is believed to occur mainly in lipid rafts [70-74]. Nevertheless, controversies exist regarding whether lipid raft localization of CD4 and the receptors is necessary for entry and productive viral replication [75,76]. Regardless of its virological significance, CD4-binding beads induced lipid raft recruitment of CXCR4, which was suggested to be actin-dependent as cytochalasin D (CytoD) partially inhibited CXCR4 clustering [74]. This observation seems to resonate with an earlier observation by Iyenger *et al.*[54], showing that HIV-1 entry can be inhibited by treatment with CytoD. Specifically, CytoD treatment prevents gp120-induced CD4/CXCR4 colocalization [54]. A remaining issue with the study is that it is not clear why actin needs to be involved when CD4 and CXCR4 are naturally associated in most cases. In addition, it is unknown whether actin is actively involved in driving the receptor clustering process or actin activity is simply an outcome following receptor clustering and signaling.

Binding of HIV particles to resting CD4 T cells was shown to trigger transient actin polymerization [27,28,77]. Vorster and Guo *et al.* further identified that the actin polymerization process is mediated through transient activation of the LIM Domain Kinase 1, LIMK1, a cellular serine/threonine kinase responsible for phosphorylation and inactivation of the actin-depolymerizing factor, cofilin [28]. Intriguingly, the signaling appears to be transduced sequentially from two sources, one from CD4 and one from CXCR4 [28]. Indeed, it has been estimated that gp120 engagement of CD4 only lasts for about 0.2 s [78], whereas fusion takes a much longer time to occur and complete [79]. Thus, gp120 may use both CD4 and CXCR4 to initiate signaling to sustain actin activity for successful fusion. Vorster and Guo *et al.* have suggested that this actin polymerization process is required to block the internalization of CXCR4 following gp120 binding, as shRNA knockdown of LIMK1 decreases the cortical actin density and causes a significant increase in the rate of CXCR4 internalization [28]. These results suggest a model in which actin is mainly involved in the stabilization of the gp120-CD4-CXCR4 complex for fusion rather than to promote the initial receptor migration and colocalization upon gp120 binding. Consistent with this mode of action, treatment of cells with CytoB inhibited fusion mediated by the CD4-independent gp120 [80], suggesting that steps sensitive to the actin inhibitor may not involve CD4 binding or CD4-CXCR4 clustering. Additionally, in HIV gp120-mediated cell-cell fusion, blocking Abl-mediated actin activity arrested fusion at the hemifusion step [59], suggesting again that the steps affected by actin dynamics are directly related to the fusion process and, more specifically, the formation of the fusion pore. In a single-molecule analysis of gp120 interaction with CD4 and CCR5, it was found that only those CCR5 receptors in close proximity to CD4 can be engaged, since this engagement has to occur very fast when gp120 is still attached to CD4 [78]. This spatial and temporal constraint likely excludes an active role of actin activity in receptor clustering and colocalization because of the relatively slower pace of HIV-mediated actin dynamics. Thus, gp120-mediatd actin activity likely plays several key roles following receptor clustering: (1) to provide a physical support to stabilize the large gp120-CD4-coreceptor aggregates on the plasma membrane; (2) to block immediate coreceptor internalization to give sufficient time for fusion to occur and complete; and (3) to prolong coreceptor signaling for priming post entry events.

The direct signaling pathway regulating this virus-mediated actin process has been partially mapped by Vorster and Guo *et al.* to be the Rac1-PAK1/2-LIMK-Cofilin pathway in resting CD4 T cells [28]. Consistently, kinases such as IL-2-inducible T-cell Kinase (ITK), a tyrosine kinase required for SDF-1-mediated actin polarization and Rac activation, has been shown to be required for HIV entry [81]. Treatment of Jurkat T cells with gp120 leads to a low, but reproducible level of ITK activation [81], which can be inhibited by ITK inhibitors or siRNA knockdown of ITK. In ITK knockdown T cells, the actin polymerization induced by gp120-coated beads was also impaired and this correlates with the decreases in viral entry [81].

Rac1 activation has also been observed during gp120-mediated cell-cell fusion between human astroglioma U87 cells and monkey BSC40 cells [57]. Expression of dominant-negative mutant of RacN17, but not Cdc42N17 or RhoN19, eliminated syncytium formation [57]; Rac activation was also inhibited by a CCR5 antagonist [57]. These results suggest that CCR5-derived signal transduction, resulting in the activation of Rac, is required for gp120-mediated cell-cell syncytium formation. Harmon *et al.* further studied multiple signaling molecules in U87 cells and suggested that Gαq, but not Gαi or Gαs, may be required for cell-cell fusion [58]. This process may involve calcium mobilization [82] and actin regulators such as Arp2/3, as cell-cell fusion can be inhibited either by siRNAs or inhibitors of upstream regulators [59]; though, an early study found that Arp2/3 inhibition, which inhibited HIV infection, did not impact cell-cell fusion [83]. These studies emphasized the complexity of the signaling network regulating actin dynamics during cell-cell syncytium formation. They also greatly expanded the scope for examining possible signaling requirements for viral entry. It is imperative to test whether similar molecules are indeed activated and specifically involved in HIV entry into its natural target cells.

In addition to direct actin regulators, recent studies have also implicated a number of actin-binding adaptor proteins and crosslinkers. For instance, Jiménez-Baranda *et al.* identified filamin-A as a cofactor for HIV entry [55]. Filamin-A, an actin-crosslinker and adaptor, was also shown to bind CD4, CCR5, and CXCR4. The authors concluded that filamin-A might be required for stability of the fusion complex. Similarly, Naghavi *et al.* identified moesin as a cellular factor whose overexpression blocks HIV and MLV infection [84]. Moesin belongs to the Ezrin-Radaxin-Moesin (ERM) family of proteins that act as crosslinkers between the plasma membrane and actin filaments and are involved in actin-directed signal transduction. Intriguingly, siRNA knockdown of moesin resulted in enhanced infection [84]. This seems to be consistent with the proposal that the cytoskeleton itself is a barrier for viral infection, and slightly disrupting the stability of actin network through actin modulators or spinoculation enhances viral infection [27-29]. Barrero-Villar *et al.* further studied the role of ERM proteins in HIV infection, and suggested that binding of gp120 to CD4 alone increases ezrin and moesin phosphorylation that might be involved in active receptor clustering [56]. In addition, siRNA knockdown of moesin, but not ezrin, resulted in an inhibition of infection. Once again, this phenomenon was attributed to an effect in receptor clustering and entry; specifically, that the association and clustering of CD4/CXCR4 induced by gp120 requires moesin-mediated anchoring of actin to the plasma membrane. Nevertheless, moesin knockdown-mediated HIV inhibition was in contrast to the enhancement observed by Naghavi *et al.*[84]. This difference could result from different cell lines used or the degrees of siRNA silencing that may affect viral processes differently.

**Figure 1:**
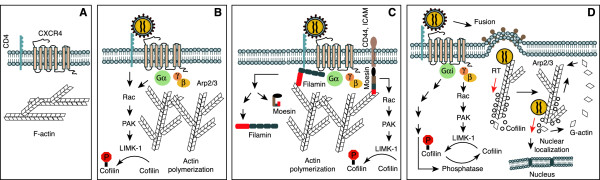
** Model of actin dynamics in HIV-1 infection of T cells.** Binding of gp120 to CD4 T cells activates Rac-PAK-LIMK-cofilin pathway, triggering early actin polymerization, transiently blocking CXCR4 internalization. Actin-anchoring proteins such as moesin and filamin-A may also serve to anchor the cortical actin to the plasma membrane to facilitate fusion. Following entry, the viral preintegration complex is directly anchored onto the cortical actin for reverse transcription and intracellular migration, which is promoted by cofilin-mediated actin treadmilling.

In summary, from the studies discussed above, we propose a prototype model in which the role of actin dynamics in HIV entry is described in Figure 1A to 1C: (1) binding of HIV gp120 to target T cells initiates a transient course of actin polymerization which is mediated through CD4/CXCR4 signal transduction that activates Pyk2, ITK and downstream Rac1, PAK1/2, LIMK1 and cofilin. (2) In the meantime, CD4 signaling or CD4/CXCR4 signaling also activates actin-anchoring proteins, such as moesin and filamin-A, to anchor F-actin to the plasma membrane and to CD4 and CXCR4. (3) Actin polymerization provides a physical support to stabilize the large gp120-CD4-CXCR4 aggregates on the plasma membrane; (4) F-actin also functions to block immediate CXCR4 internalization to give sufficient time for fusion to occur and complete. (5) Additionally, actin-supported stability of the gp120-CD4-CXCR4 complex may prolong coreceptor signaling for priming postentry events.

### Role of actin in HIV-1 postentry DNA synthesis

Following viral fusion and entry, HIV goes through the processes of uncoating and reverse transcription. The involvement of the actin cytoskeleton in viral postentry steps, particularly in the process of reverse transcription, remains poorly characterized. Early indications that components of HIV particles may interact with the cytoskeleton came from studies in 1992 by Arthur *et al.* who identified actin, among other cellular proteins, in virions collected from sucrose gradient centrifugation [85]. Ott *et al.* also found three types of actin-related factors present inside HIV virions in various molar ratios to HIV Gag: actin (10-15%), ezrin and moesin (2%), and cofilin (2-10%) [86,87]. The functional significance of the virion-incorporation of these proteins is unknown, although Wilk *et al.* demonstrated that the packaged actin filaments are specifically associated with the nucleocapsid (NC) [11]. Through various screening strategies such as yeast two-hybrid system, several other viral proteins have also been found to interact with actin. These include the large subunit of the reverse transcriptase [7], the integrase [13], Nef [12,14], and part of the Gag polyprotein or the nucleocapsid [8-11]. All of these proteins are integral components of the viral preintegration complex (PIC) delivered into cells. These findings seem to suggest that actin may play a role in defining the structure of the virion particle or the PIC. Whether this actin-supported structural integrity is important for viral postentry process such as reverse transcription has not been clearly resolved.

There is some indication that a direct interaction with the actin cytoskeleton following viral entry may be unnecessary or avoidable. Firstly, reverse transcription can be initiated from permeabilized virion particles [88,89], and naturally, HIV particles also contain partially reverse transcribed genomes [90-92]. Secondly, pseudotyping HIV particles with VSV-G, which mediates viral entry through endocytosis, generates highly infectious virus that infects cells with minimal contact with the cortical actin [15,17,93,94]. Nevertheless, the DNA molecules found in virion particles are mostly early products of reverse transcription [90,91], and the process of intravirion DNA synthesis is also inefficient; the ratio of genomic RNA to viral DNA is very low, at 10^3^:1 for the early “strong stop” DNA and 10^5^:1 for the late “*gag*” DNA [89]. These findings are somewhat reminiscent of the reverse transcription reaction using purified reverse transcriptase, in which only the early product of the reverse transcription can be generated [95]. These data suggest that there may be structural constraints in the virion, limiting reverse transcription; alternatively, cellular cofactors such as actin or actin-associated factors may be required. Intriguingly, intravirion reverse transcription can be stimulated using deoxyribonucleoside triphosphates (dNTPs) and detergent to partially disrupt virion structure [89,96,97]. These detergent-induced changes include dissolution of the p24 shell in the viral core and disappearance of the core-envelope linkage region [98]. Based on these findings, Zhang *et al.* suggested that intravirion or intracellular reverse transcription of HIV-1 is unlikely to take place within intact viral cores [98]. Thus, effective viral reverse transcription inside cells may involve an organized process of uncoating or core rearrangement following entry [99-101].

In VSV-G pseudotyped HIV infection, the role of cortical actin in VSV-G-mediated endocytosis is limited to membrane scission of clathrin-coated pits, which does not involve direct contact between the cortical actin and viral particles [102]. The higher infectivity of VSV-G pseudotyped virus could suggest that HIV uncoating or reverse transcription may not need the extensive involvement of the cortical actin in cells. However, Yu *et al.* demonstrated that, though nuclear migration is more efficient following VSV-G-mediated entry, viral DNA synthesis is decreased [19]. These data suggest that the higher infectivity of the VSV-G pseudotyped particles does not result from higher levels of viral entry, uncoating or reverse transcription, but is largely attributed to the greater ability of VSV-G to deliver the PIC deep into the cytoplasm for nuclear entry. On the other hand, the particles generated by VSV-G pseudotyping may be unnatural or imperfectly formed, especially in the core-envelope linkage region [15,98]. Thus, the uncoating process during VSV-G-mediated endocytic entry may not be as tightly regulated as in the natural HIV particle. Indeed, Brun *et al.* demonstrated that certain CA mutants that affect core assembly and stability, though defective during wild-type HIV replication, could be rescued by pseudotyping with VSV-G [103]. These results indicate that proper core structure is required for uncoating, reverse transcription and nuclear import when the core is delivered by fusion at the plasma membrane; however, such a requirement is dispensable when the core is delivered through the endocytic pathway. Therefore, the VSV-G-mediated infection may not reflect the genuine uncoating requirements, which may require cortical actin or cortical actin-associated factors. In addition, both Agosto *et al.*[20] and Yu *et al.*[19] demonstrated that, although highly infectious in transformed cells, the VSV-G pseudotyped virus is not capable of infecting resting CD4 T cells, as viral DNA synthesis is highly diminished [19]. These results, although they do not demonstrate a direct role of cytoskeletal actin in HIV reverse transcription, do suggest that the cortical actin may be involved in the process of uncoating and/or reverse transcription, especially in HIV infection of its natural target cells.

The process of HIV uncoating is poorly studied [104], and there is no specific cellular cofactor identified so far, although there are some suggestions that cellular kinases may be involved [101]. Additionally, it has also been suggested that activated T cells may contain an uncoating factor, whereas resting CD4 T cells do not [105]. Given that the cortical actin is the immediate structure intercepting the virus after membrane fusion, there is a possibility that actin-associated factors or actin activity itself may be a direct driving force for uncoating. The mechanical force generated by actin polymerization and exerted by the lamellipodium on a moving cell has been measured to be in the range of a few nanonewtons (nN) [106,107]. It would be interesting to know whether the actin forces triggered during HIV entry are sufficient to destabilize the core structure.

Some of the direct evidence demonstrating a requirement for cytoskeletal actin in HIV reverse transcription came from multiple inhibitor studies as well as recent siRNA knockdown studies of the actin modulators. Iyengar *et al.* demonstrated that CytoD treatment (0.2 to 1 μM) of PHA-activated peripheral blood mononuclear cells (PBMC) led to inhibition of HIV infection [54]. Nevertheless, the authors concluded that the inhibitory effect of CytoD resulted from specific blockage of viral entry and CD4 and CXCR4 receptor clustering [54]. Bukrinskaya *et al.* also reported that CytoA, CytoD, and CytoE (5 μM) all inhibited HIV infection of a HeLa-CD4 indicator cell [108]. Specifically, CytoD reduced viral early DNA synthesis 4- to 5-fold in MT-4 cells [108]. The study did not distinguish whether the decrease of viral DNA resulted from a loss of entry as proposed by Iyengar [54]. However, the authors argued that the inhibitory effects of cytochalasins were mainly from inhibiting viral DNA synthesis rather than from inhibiting entry, since early viral DNA was abundantly detected in treated cells [108].

Yoder *et al.* also reported that the actin inhibitor jasplakinolide (Jas) effectively inhibited HIV latent infection of resting CD4 T cells at dosages around 120 nM when T cell activation was not inhibited by Jas [27]. The inhibition was largely attributed to the inhibition of viral DNA synthesis and nuclear migration, although viral entry was also slightly inhibited by 120 nM Jas [27]. Guo *et al.* further demonstrated that the Jas inhibition of HIV infection was also dependent on cellular states [29]. For resting CD4 T cells, in which the cytoskeletal actin is relatively static [27], the Jas IC50 dosage for HIV-1 infection was 60 nM, whereas for transformed Rev-CEM indicator cells [109,110], the Jas IC_50_ for HIV-1 infection was 250–500 nM [29]. In addition, increasing actin dynamics through spinoculation increased the IC_50_ to approximately 1 μM for Rev-CEM cells [29]. These results demonstrate that increasing actin dynamics can partially overcome the Jas inhibitory effect on HIV infection. Guo *et al.* further suggested that the relative sensitivity to Jas could be used as a correlative of actin dynamics in different cell types, and hence, part of their capacity to support HIV infection [29].

A major caveat of using actin inhibitors to study HIV infection is that most of them are associated with general cytotoxicity and have broad effects on cell physiology. Thus, proper controls need to be used to ensure that the inhibition observed is virus specific. Nevertheless, these actin inhibitor studies do appear to be consistent with several siRNA knockdown studies in which the activity of actin modulators such as cofilin and LIMK1 was directly targeted. Yoder *et al.* demonstrated that slight shRNA-mediated inhibition of cofilin, an actin-depolymerizing factor, in pre-activated blood CD4 T triggered a drastic increase in the cortical actin density [27]; however, similar knockdown of cofilin in transformed CEM-SS T cells triggered apoptosis [27]. Even in the background of potential cytotoxicity, the increase in the cortical actin in blood CD4 T cells correlated with an increase in HIV DNA synthesis [27]. Consistently, shRNA knockdown of the cofilin kinase LIMK1 decreased the cortical actin density, yet increased CXCR4 receptor trafficking [28]. In the LIMK1 knockdown cells, a decrease in cortical actin was associated with an impairment of viral DNA synthesis following infection with wild type HIV-1, but not with the VSV-G pseudotyped virus [28]. These results demonstrate a direct role of the cortical actin in early viral DNA synthesis. However, these actin inhibitor and siRNA knockdown studies do not provide a mechanistic understanding of why altering actin density or dynamics affect HIV DNA synthesis. Vorster and Guo *et al.* suggest that the process of actin polymerization may simply act as a driving force for viral uncoating, and thus, decreasing actin dynamics may impair proper uncoating [28]. Alternatively or additionally, the cortical actin may function as an anchorage for the viral reverse transcription complex, and a decrease in the actin cortex density may result in less contact time and suboptimal reverse transcription [28]. Bukrinskaya *et al.* has also demonstrated that phosphorylated Gag matrix protein (MA) is mainly associated with the HIV preintegration complex (PIC) and with the actin cytoskeleton during early stages of HIV infection, and suggested that actin filaments may be a major site for viral reverse transcription in infected cells [108]. Others have demonstrated that the Gag nucleocapsid protein (NC) rather than MA is the factor that directly interacts with actin [8-11]. Certainly, besides Gag NC, multiple HIV proteins such as the large subunit of the viral reverse transcriptase, the viral integrase, and Nef in the preintegration complex (PIC) are known to directly interact with actin [7,12-14], suggesting possible anchorage of PIC onto the cortical actin for efficient reverse transcription. Among these actin interacting proteins, Nef in particular has been known to enhance viral infectivity by a factor of 4 to 40 [111-113]. This enhancement has been attributed to a positive effect of Nef at early steps postentry, such as uncoating or reverse transcription [114-116]. Nef has been known to also interact with the HIV core [117,118] and requires cellular cofactors for enhancement, since Nef-defective virions display normal levels of endogenous reverse transcriptase activity [116]. This positive effect of Nef in infected cells is diminished by actin inhibitors [18] or pseudotyping with VSV-G [15]. These results indicate that the Nef-mediated enhancement of viral DNA synthesis is likely related to the cortical actin.

In HIV infection of resting CD4 T cells, viral DNA synthesis is a slow process that takes about 2 days to maximize [27]. After reaching its peak level, viral DNA decreases with time, likely resulting from a concurrent decay process [27,119]. Korin and Zack suggested that a lower dNTP level in resting CD4 T cells may limit viral DNA synthesis [120,121]. Yoder and Yu *et al.* demonstrated that the slower synthesis and decay of viral DNA are also correlated with lower cortical actin dynamics in resting T cells [27]. To increase actin dynamics, the virus utilizes gp120 binding to CXCR4 to trigger a transient course of actin activity through LIMK1 and cofilin [27,28,32]. This actin process facilitates viral DNA synthesis and nuclear migration, which may mitigate the viral DNA decay process. As early as 2 h post infection, a significant fraction of viral DNA is translocated into the nucleus, concurrent with HIV-mediated signaling and actin activity [27]. HIV DNA synthesized in the absence of actin activity may be excluded from nuclear entry and gradually degraded in the cytoplasm of resting T cells [27]. Consistently, pre-stimulation of resting CD4 T cells with anti-CD4/CXCR4 beads reorganized the cortical actin and increased actin dynamics, and this stimulation enhanced viral DNA synthesis and nuclear migration [27]. In addition, Guo *et al.* demonstrated that spinoculation triggered dynamic actin and cofilin activity, and this process dramatically increased viral DNA synthesis and nuclear migration in resting CD4 T cells [29]. Furthermore, Campbell *et al.* demonstrated that stimulation of resting CD4 T cells with CCL2 augmented gp120-induced F-actin polymerization, which enhanced viral DNA synthesis about 5-fold [122]. These results are in agreement with a model in which actin may be involved in viral uncoating and DNA synthesis. In resting T cells, a lessened amount of actin activity may be associated with a slower uncoating rate, which could result in a slower course of viral DNA synthesis and nuclear migration and an increased rate of viral DNA decay.

In summary, based on the above discussion, a possible role of the cortical actin in viral postentry DNA synthesis is described in Figure 1D. Following viral fusion and entry, HIV-mediated actin polymerization may facilitate viral uncoating; the viral preintegration complex may also be directly anchored onto actin filaments through multiple proteins such as the viral nucleocapsid, the large subunit of RT, integrase, and Nef. The anchorage of the preintegration complex onto actin filaments may be important for optimal reverse transcription and subsequent nuclear migration.

### Role of actin in HIV intracellular migration

After, or concomitant with reverse transcription, the viral preintegration complex (PIC) must migrate to the nucleus for integration. The PIC retains many of the viral factors associated with reverse transcription; most notably, the reverse transcriptase, the integrase, the matrix, the nucleocapsid, Vif, Vpr, and Nef [123]. Among these PIC components, the large subunit of the reverse transcriptase, the integrase, the nucleocapsid, and Nef are also known to interact with actin [7-14]. Until recently, there has been very little insight into the possible role of actin in PIC intracellular migration and delivery to the nuclear peripheral zone; however, recent evidence seems to suggest that, once again, actin may play a pivotal role in this essential infective process.

Live cell imaging of GFP-tagged HIV-1 by McDonald *et al.*[124] revealed microtubule-associated intracellular motility in HeLa and Hos cells, which could be inhibited by microinjection of an anti-dynein motor complex antibody. By 2 h post-infection, a significant proportion of the labeled particles accumulated in the perinuclear region, often at the microtubule-organizing center (MTOC). Though microtubule or F-actin inhibition alone did not inhibit intracellular migration, a combination of nocodazole and latrunculin B, which disrupt microtubule and actin polymerization respectively, did diminish particle motility [124], suggesting that this process may involve both microtubules and actin. Similarly, Arhel *et al.*, used labeled integrase to track the intracellular movements of VSV-G-pseudotyped HIV-1 in HeLa cells, and suggested that both microtubule- and actin-dependent movements may be involved in HIV migration [125]. Treatment of cells with a dominant-negative dynactin or latrunculin B impaired viral cytosolic movement [125].

Given that a majority of particles in a viral preparation are often not infectious [126] and funneled for degradation after entry [127,128], a cautionary note has been made that these and similar imaging studies [129] may not distinguish replication-competent viruses from the non-infectious particles or particle aggregates, which represent the majority [130]. Yoder *et al.* used multiple microtubule modulators such as taxol, vinblastine, colchicine, and nocodazole to provide direct biological evidence for the involvement of microtubules in early steps of HIV infection of CD4 T cells [131]. However, the authors observed almost no inhibition of HIV-1 infection, although these drugs disrupted microtubule integrity. These results do not appear to support an essential role of microtubules in the initiation of HIV infection of CD4 T cells. In contrast, Yoder *et al.* observed effective inhibition of HIV latent infection of blood CD4 by the actin inhibitor jasplakinolide at 120 nM [131]. The authors postulated that CD4 T cells do not have an extensive microtubule network as do HeLa cells, and that the relative thin cytoplasm in T cells may require only actin-based, short-distance travel for nuclear localization.

Yoder and Yu *et al.* furthered this line of evidence supporting a critical role of the actin cytoskeleton in HIV-1 infection and nuclear migration [27]. The authors suggest that the cortical actin in resting blood CD4 T cells is relatively static in the absence of T cell activation or chemotactic stimulation. This lack of actin activity may represent a realistic limitation for viral early processes such as entry and intracellular migration. To overcome this limitation, HIV uses the chemokine receptor CXCR4 to trigger the activation of actin modulators such as LIMK1 and cofilin to increase actin dynamics [27,28]. Indeed, the authors demonstrated that inhibition of CXCR4-associated Gαi signaling with pertussis toxin inhibited HIV-mediated cofilin activation and actin dynamics, which resulted in a decrease of viral nuclear DNA as early as 2 h post infection [27]. Similarly, treatment of cells with jasplakinolide diminished both HIV-1 DNA synthesis and nuclear migration [27]. In addition, knockdown of cofilin induced a marked increase in the cortical actin density, which enhanced early HIV DNA synthesis while hindering HIV nuclear migration [27]. These findings were corroborated using supplemental methodologies for increasing actin dynamics. For instance, treatment of cells with anti-CD4/CXCR4 antibody-conjugated magnetic beads, which mimic viral binding and signaling, greatly stimulates actin dynamics and viral early DNA synthesis and nuclear migration [27]. Induction of actin dynamics through transient treatment with latrunculin A or a cofilin-activating peptide also enhanced HIV latent infection of CD4 T cells [27]. These findings suggest that actin dynamics play a critical role in viral DNA synthesis and nuclear migration in the infection of resting T cells. Furthermore, these findings also demonstrate that HIV actively promotes actin activity through exploiting the chemokine signaling network by choosing the chemokine receptors, CXCR4 and CCR5, as the co-receptors for binding and entry [31].

Yu *et al.* supported these findings in a subsequent study regarding the potential role of signaling and actin in the infection of primary CD4 T cells [19]. As per previous studies, VSV-G-pseudotyped HIV-1, which enters cells through endocytosis, exhibits a more efficacious infection in transformed and activated T cells [15,17]. However, this effect was not recapitulated in latent infection of resting T cells: Only the HIV envelope-mediated entry, but not the VSV-G-mediated endocytosis, can lead to viral DNA synthesis and nuclear migration [19]. The viral particles entering through the endocytic pathways were destroyed within 1–2 days. Notably, these findings indicate that the pH-dependent route of entry may not be a viable mechanism in primary resting CD4 T cells [21]; the capacity of HIV gp120 to trigger signaling and to engage the cortical actin seems to be critical for viral postentry stability. Though, this study does not directly lend evidence for the role of actin in viral nuclear migration, within the broader contexts of accumulated findings, this extrapolation is most consistent with that model.

A study by Cameron and Saleh *et al.* furthered the nascent model regarding the roles of chemokine signaling and actin dynamics in HIV-1 latent infection of blood CD4 T cells [30]. This study focused on the role of chemokines—especially CCL19—in HIV-1 latent infection of resting T cells: Pre-treatment of resting CD4 T cells with the chemokines CCL19, CXCL9, CXCL10, and CCL20 led to a significant increase in integrated viral DNA [30,132,133]. More detailed analysis of CCL19 indicated that the mechanism was associated with CCL19-mediated cofilin activation and changes in actin filaments, as the CCL19-mediated enhancement of viral nuclear localization and integration was inhibited by the actin inhibitor jasplakinolide [30]. Cumulatively, these findings suggest that chemokine-mediated actin dynamics play a pivotal role in HIV-1 nuclear migration, and the establishment of latent infection of resting T cells [33].

A recent study by Vorster and Guo *et al.* further identified the key signaling event involved in HIV-mediated reorganization of actin filaments in resting T cells [28]. In this study, it was shown that HIV-1 gp120 led to actin dynamics in a manner that correlated with the activation of the Rac-PAK-LIMK-cofilin pathway. Brief treatment of resting T cells with okadaic acid, a non-specific LIMK activator and phosphatase inhibitor, triggered dramatic LIMK activation and actin polymerization, which led to a significant increase in HIV-1 latent infection. Furthermore, shRNA-mediated knockdown of LIMK1 decreased filamentous actin, increased surface CXCR4 trafficking, and diminished viral DNA synthesis and nuclear migration. These results support the model in which HIV-mediated early actin dynamics directly regulates the CXCR4 receptor during viral entry and is involved in viral DNA synthesis and nuclear migration.

The above study also stimulated Guo *et al.* to postulate that the centrifugal force generated during spinoculation, a technique commonly used to enhance viral infectivity through low speed spinning of cells (1,000 – 2,000 x g) [134-138], may induce stress-related signaling and actin dynamics [29]. Indeed, spinoculation was found to trigger both cofilin activation and actin dynamics in transformed and resting CD4 T cells [29]. This led to the upregulation of CXCR4 and a great enhancement of HIV-1 DNA synthesis and nuclear migration, which can be inhibited by jasplakinolide and shRNA knockdown of LIMK1 [29]. These results highlight the importance of cofilin and a dynamic actin cytoskeleton in the initiation of HIV infection.

Based on these recent studies, the role of the cortical actin in viral postentry migration in CD4 T cells is summarized in Figure 1D. At the early time following HIV entry, concurrent with the anchorage of the PIC onto actin filaments for reverse transcription, HIV-mediated cofilin activation stimulates actin treadmilling, which may carry the PIC across the cortical actin. Further traveling of the PIC to the perinuclear region may continue to rely on actin filaments or some viral factors such as Nef to trigger localized actin activity.

### Relationship between HIV-mediated receptor signaling and actin dynamics

While it is increasingly clear that cytoskeletal actin is required for viral infection, the relationship between HIV-mediated receptor signaling and actin dynamics is not straightforward. Chemokine receptor signaling normally leads to actin dynamics for driving cell migration. However, multiple previous studies have demonstrated that chemokine coreceptor signaling is not required for viral entry or replication in transformed cell lines [139-148]. For example, Farzan *et al.* created three CCR5 mutants that abolished their signaling ability to mobilize calcium, but detected minimal effects on viral entry or replication [140]. Consistently, CXCR4 mutations that eliminate G protein-coupled signaling show no inhibition of HIV entry and replication [146,147]; for instance, truncation of the C-terminal tail of CXCR4 or CCR5 effectively blocked calcium flux, but did not affect HIV entry or replication [141,142,144,145]. This is in great contrast to the extensive dependency on signaling molecules observed during cell-cell fusion [59] or HIV-1 infection of resting CD4 T cells [27,28], which appears to require a different signaling environment.

Yoder and Yu *et al.* have given an explanation regarding the conflicting observation between viral requirement for actin dynamics and the dispensable role of chemotactic signaling for HIV infection of transformed cells [27]. The authors suggested that in transformed cell lines, the cell cycle takes control of actin dynamics and HIV-mediated signal transduction to the actin cytoskeleton may be diverted or become secondary. This is particularly reflected in HIV infection of transformed *versus* resting blood CD4 T cells. Yoder and Yu *et al.* demonstrated that treatment of resting T cells with HIV particles triggers a transient course of actin activity, indicative of active signal transduction to the actin cytoskeleton; whereas similar treatment of transformed CEM-SS T cells did not trigger any measurable actin activity [27], suggestive of reduced or derailed signals to the actin cytoskeleton. Thus, in cycling cells, while targeting signal transduction from the chemokine coreceptor may not inhibit HIV, directly targeting actin or actin modulators such as cofilin, LIMK, Rac1, WAVE2, and Arp2/3 [27,29,59] would lead to the inhibition of actin dynamics. This may specifically or non-specifically impact HIV entry or early postentry processes depending on whether the actual activation of signaling molecules occurs in response to HIV binding.

Given the fundamental need for HIV to interact with the actin cytoskeleton, the virus may redundantly use multiple resources to engage the actin network. The virus may use both CD4 and the chemokine coreceptors to trigger actin dynamics [28]. In addition, HIV may transduce signals leading to actin dynamics from both Gαi [27] and Gαq [58]. Thus, inhibition of signal transduction from one receptor may not inhibit similar signals from the other. This signaling redundancy may be an evolved functionality of gp120 to ensure that the critical requirements for actin dynamics are met early.

## Conclusions

Recent findings have begun to highlight actin and actin dynamics in HIV replication [149,150]: Actin activity has been shown to be important for viral entry, either for receptor dynamics or stabilization of the fusion complex; actin may additionally be necessary for proper uncoating and efficient reverse transcription; actin dynamics and actin regulators such as LIMK and cofilin are required for efficient nuclear migration in primary target cells such as resting CD4 T cells. In addition, although beyond the scope of this review, actin may be involved in HIV cell-cell transmission [151-153] and viral assembly and the budding process [86,87,152,154-157]. Furthermore, it has also been demonstrated recently that signal transduction from chemokines such as CCL2, CCL19, and CCL21 increases the permissiveness of resting T cells to HIV-1 through induction of actin dynamics [33,122,132,158-160]. These studies not only shed light on the molecular aspects of the HIV life cycle, but also have implications in viral pathogenesis. It is possible that HIV- or viral protein-mediated disruption of actin regulatory network may contribute to viral pathogenesis through disruption of normal chemotactic responses and T cell activity [31,32,161].

Pharmacologically, targeting the actin cytoskeleton may not be practical because of the known cytotoxicity of actin drugs and the need for long-term treatment. However, by interfering with the chemokine receptors or the downstream signaling molecules, a less pervasive disruption of HIV-mediated actin activity is achieved. Approximately 50% of all marketed therapeutic drugs target G protein-coupled receptors such as the ones HIV uses [162]. As such, a much-needed future focus should be on the illumination of signal transduction events associated with HIV infection and HIV-mediated actin activity, particularly in the primary target cells of HIV. Any so-discovered cellular protein, ideally one of narrow tissue distribution and function, could be a hypothetical target for therapeutic intervention with acceptable adverse effects.

## Competing interests

The authors declare that they have no competing interest.

## Authors’ contributions

YW conceived the manuscript and was responsible for organizing the content. YW, MS, JG wrote the manuscript. YW was responsible for creating Figure 1 and the model. All authors read and approved the final manuscript.

## References

[B1] Pollard TD Borisy GG Cellular motility driven by assembly and disassembly of actin filaments Cell 2003 112 453 465 10.1016/S0092-8674(03)00120-X 12600310

[B2] Loisel TP Boujemaa R Pantaloni D Carlier MF Reconstitution of actin-based motility of Listeria and Shigella using pure proteins Nature 1999 401 613 616 10.1038/44183 10524632

[B3] Cameron LA Giardini PA Soo FS Theriot JA Secrets of actin-based motility revealed by a bacterial pathogen Nat Rev Mol Cell Biol 2000 1 110 119 10.1038/35040061 11253363

[B4] Frischknecht F Moreau V Rottger S Gonfloni S Reckmann I Superti-Furga G Way M Actin-based motility of vaccinia virus mimics receptor tyrosine kinase signalling Nature 1999 401 926 929 10.1038/44860 10553910

[B5] Goley ED Ohkawa T Mancuso J Woodruff JB D'Alessio JA Cande WZ Volkman LE Welch MD Dynamic nuclear actin assembly by Arp2/3 complex and a baculovirus WASP-like protein Science 2006 314 464 467 10.1126/science.1133348 17053146

[B6] Ohkawa T Volkman LE Welch MD Actin-based motility drives baculovirus transit to the nucleus and cell surface Journal of Cell Biology 2010 190 187 195 10.1083/jcb.201001162 20660627 PMC2930276

[B7] Hottiger M Gramatikoff K Georgiev O Chaponnier C Schaffner W Hubscher U The large subunit of HIV-1 reverse transcriptase interacts with beta-actin Nucleic Acids Res 1995 23 736 741 10.1093/nar/23.5.736 7535922 PMC306752

[B8] Rey O Canon J Krogstad P HIV-1 Gag protein associates with F-actin present in microfilaments Virology 1996 220 530 534 10.1006/viro.1996.0343 8661406

[B9] Ibarrondo FJ Choi R Geng YZ Canon J Rey O Baldwin GC Krogstad P HIV type 1 Gag and nucleocapsid proteins: cytoskeletal localization and effects on cell motility AIDS Res Hum Retroviruses 2001 17 1489 1500 10.1089/08892220152644197 11709093

[B10] Liu B Dai R Tian CJ Dawson L Gorelick R Yu XF Interaction of the human immunodeficiency virus type 1 nucleocapsid with actin J Virol 1999 73 2901 2908 10074138 10.1128/jvi.73.4.2901-2908.1999 PMC104048

[B11] Wilk T Gowen B Fuller SD Actin associates with the nucleocapsid domain of the human immunodeficiency virus Gag polyprotein J Virol 1999 73 1931 1940 9971772 10.1128/jvi.73.3.1931-1940.1999 PMC104434

[B12] Fackler OT Kienzle N Kremmer E Boese A Schramm B Klimkait T Kucherer C Mueller-Lantzsch N Association of human immunodeficiency virus Nef protein with actin is myristoylation dependent and influences its subcellular localization Eur J Biochem 1997 247 843 851 10.1111/j.1432-1033.1997.00843.x 9288906

[B13] Turlure F Devroe E Silver PA Engelman A Human cell proteins and human immunodeficiency virus DNA integration Front Biosci 2004 9 3187 3208 10.2741/1472 15353349

[B14] Niederman TM Hastings WR Ratner L Myristoylation-enhanced binding of the HIV-1 Nef protein to T cell skeletal matrix Virology 1993 197 420 425 10.1006/viro.1993.1605 8212577

[B15] Aiken C Pseudotyping human immunodeficiency virus type 1 (HIV-1) by the glycoprotein of vesicular stomatitis virus targets HIV-1 entry to an endocytic pathway and suppresses both the requirement for Nef and the sensitivity to cyclosporin A J Virol 1997 71 5871 5877 9223476 10.1128/jvi.71.8.5871-5877.1997 PMC191842

[B16] Chazal N Singer G Aiken C Hammarskjold ML Rekosh D Human immunodeficiency virus type 1 particles pseudotyped with envelope proteins that fuse at low pH no longer require Nef for optimal infectivity J Virol 2001 75 4014 4018 10.1128/JVI.75.8.4014-4018.2001 11264394 PMC114896

[B17] Luo T Douglas JL Livingston RL Garcia JV Infectivity enhancement by HIV-1 Nef is dependent on the pathway of virus entry: implications for HIV-based gene transfer systems Virology 1998 241 224 233 10.1006/viro.1997.8966 9499797

[B18] Campbell EM Nunez R Hope TJ Disruption of the actin cytoskeleton can complement the ability of Nef to enhance human immunodeficiency virus type 1 infectivity Journal of Virology 2004 78 5745 5755 10.1128/JVI.78.11.5745-5755.2004 15140972 PMC415815

[B19] Yu D Wang W Yoder A Spear M Wu Y The HIV envelope but not VSV glycoprotein is capable of mediating HIV latent infection of resting CD4 T cells PLoS Pathog 2009 5 e1000633 10.1371/journal.ppat.1000633 19851458 PMC2760144

[B20] Agosto LM Yu JJ Liszewski MK Baytop C Korokhov N Humeau LM O'Doherty U The CXCR4-tropic human immunodeficiency virus envelope promotes more-efficient gene delivery to resting CD4+ T cells than the vesicular stomatitis virus glycoprotein G envelope J Virol 2009 83 8153 8162 10.1128/JVI.00220-09 19493998 PMC2715791

[B21] Miyauchi K Kim Y Latinovic O Morozov V Melikyan GB HIV enters cells via endocytosis and dynamin-dependent fusion with endosomes Cell 2009 137 433 444 10.1016/j.cell.2009.02.046 19410541 PMC2696170

[B22] Stein BS Gowda SD Lifson JD Penhallow RC Bensch KG Engleman EG pH-independent HIV entry into CD4-positive T cells via virus envelope fusion to the plasma membrane Cell 1987 49 659 668 10.1016/0092-8674(87)90542-3 3107838

[B23] Maddon PJ McDougal JS Clapham PR Dalgleish AG Jamal S Weiss RA Axel R HIV infection does not require endocytosis of its receptor, CD4 Cell 1988 54 865 874 10.1016/S0092-8674(88)91241-X 3261635

[B24] McClure MO Marsh M Weiss RA Human immunodeficiency virus infection of CD4-bearing cells occurs by a pH-independent mechanism Embo J 1988 7 513 518 3259178 10.1002/j.1460-2075.1988.tb02839.x PMC454348

[B25] Brandt SM Mariani R Holland AU Hope TJ Landau NR Association of chemokine-mediated block to HIV entry with coreceptor internalization J Biol Chem 2002 277 17291 17299 10.1074/jbc.M108232200 11782464

[B26] Pelchen-Matthews A Clapham P Marsh M Role of CD4 endocytosis in human immunodeficiency virus infection J Virol 1995 69 8164 8168 7494343 10.1128/jvi.69.12.8164-8168.1995 PMC189775

[B27] Yoder A Yu D Dong L Iyer SR Xu X Kelly J Liu J Wang W Vorster PJ Agulto L HIV envelope-CXCR4 signaling activates cofilin to overcome cortical actin restriction in resting CD4 T cells Cell 2008 134 782 792 10.1016/j.cell.2008.06.036 18775311 PMC2559857

[B28] Vorster PJ Guo J Yoder A Wang W Zheng Y Xu X Yu D Spear M Wu Y LIM kinase 1 modulates cortical actin and CXCR4 cycling and is activated by HIV-1 to initiate viral infection J Biol Chem 2011 286 12554 12564 10.1074/jbc.M110.182238 21321123 PMC3069457

[B29] Guo J Wang W Yu D Wu Y Spinoculation triggers dynamic actin and cofilin activity facilitating HIV-1 infection of transformed and resting CD4 T cells J Virol 2011 85 9824 9833 10.1128/JVI.05170-11 21795326 PMC3196392

[B30] Cameron PU Saleh S Sallmann G Solomon A Wightman F Evans VA Boucher G Haddad EK Sekaly RP Harman AN Establishment of HIV-1 latency in resting CD4+ T cells depends on chemokine-induced changes in the actin cytoskeleton Proc Natl Acad Sci U S A 2010 107 16934 16939 10.1073/pnas.1002894107 20837531 PMC2947912

[B31] Wu Y Yoder A Chemokine coreceptor signaling in HIV-1 infection and pathogenesis PLoS Pathog 2009 5 e1000520 10.1371/journal.ppat.1000520 20041213 PMC2790611

[B32] Wu Y The co-receptor signaling model of HIV-1 pathogenesis in peripheral CD4 T cells Retrovirology 2009 6 41 10.1186/1742-4690-6-41 19409100 PMC2679705

[B33] Wu Y Chemokine control of HIV-1 infection: beyond a binding competition Retrovirology 2010 7 86 10.1186/1742-4690-7-86 20942936 PMC2964589

[B34] Bukrinsky M How to engage Cofilin Retrovirology 2008 5 85 10.1186/1742-4690-5-85 18808680 PMC2567344

[B35] Liu Y Belkina NV Shaw S HIV infection of T cells: actin-in and actin-out Sci Signal 2009 2 pe23 10.1126/scisignal.266pe23 19366992

[B36] Klatzmann D Barre-Sinoussi F Nugeyre MT Danquet C Vilmer E Griscelli C Brun-Veziret F Rouzioux C Gluckman JC Chermann JC Selective tropism of lymphadenopathy associated virus (LAV) for helper-inducer T lymphocytes Science 1984 225 59 63 10.1126/science.6328660 6328660

[B37] Klatzmann D Champagne E Chamaret S Gruest J Guetard D Hercend T Gluckman JC Montagnier L T-lymphocyte T4 molecule behaves as the receptor for human retrovirus LAV Nature 1984 312 767 768 10.1038/312767a0 6083454

[B38] Dalgleish AG Beverley PC Clapham PR Crawford DH Greaves MF Weiss RA The CD4 (T4) antigen is an essential component of the receptor for the AIDS retrovirus Nature 1984 312 763 767 10.1038/312763a0 6096719

[B39] McDougal JS Mawle A Cort SP Nicholson JK Cross GD Scheppler-Campbell JA Hicks D Sligh J Cellular tropism of the human retrovirus HTLV-III/LAV. I. Role of T cell activation and expression of the T4 antigen J Immunol 1985 135 3151 3162 2995487

[B40] McDougal JS Kennedy MS Sligh JM Cort SP Mawle A Nicholson JK Binding of HTLV-III/LAV to T4+ T cells by a complex of the 110 K viral protein and the T4 molecule Science 1986 231 382 385 10.1126/science.3001934 3001934

[B41] Maddon PJ Dalgleish AG McDougal JS Clapham PR Weiss RA Axel R The T4 gene encodes the AIDS virus receptor and is expressed in the immune system and the brain Cell 1986 47 333 348 10.1016/0092-8674(86)90590-8 3094962

[B42] Feng Y Broder CC Kennedy PE Berger EA HIV-1 entry cofactor: functional cDNA cloning of a seven-transmembrane, G protein-coupled receptor Science 1996 272 872 877 10.1126/science.272.5263.872 8629022

[B43] Cocchi F DeVico AL Garzino-Demo A Arya SK Gallo RC Lusso P Identification of RANTES, MIP-1 alpha, and MIP-1 beta as the major HIV-suppressive factors produced by CD8+ T cells Science 1995 270 1811 1815 10.1126/science.270.5243.1811 8525373

[B44] Alkhatib G Combadiere C Broder CC Feng Y Kennedy PE Murphy PM Berger EA CC CKR5: a RANTES, MIP-1alpha, MIP-1beta receptor as a fusion cofactor for macrophage-tropic HIV-1 Science 1996 272 1955 1958 10.1126/science.272.5270.1955 8658171

[B45] Choe H Farzan M Sun Y Sullivan N Rollins B Ponath PD Wu L Mackay CR LaRosa G Newman W The beta-chemokine receptors CCR3 and CCR5 facilitate infection by primary HIV-1 isolates Cell 1996 85 1135 1148 10.1016/S0092-8674(00)81313-6 8674119

[B46] Deng H Liu R Ellmeier W Choe S Unutmaz D Burkhart M Di Marzio P Marmon S Sutton RE Hill CM Identification of a major co-receptor for primary isolates of HIV-1 Nature 1996 381 661 666 10.1038/381661a0 8649511

[B47] Doranz BJ Rucker J Yi Y Smyth RJ Samson M Peiper SC Parmentier M Collman RG Doms RW A dual-tropic primary HIV-1 isolate that uses fusin and the beta-chemokine receptors CKR-5, CKR-3, and CKR-2b as fusion cofactors Cell 1996 85 1149 1158 10.1016/S0092-8674(00)81314-8 8674120

[B48] Dragic T Litwin V Allaway GP Martin SR Huang Y Nagashima KA Cayanan C Maddon PJ Koup RA Moore JP Paxton WA HIV-1 entry into CD4+ cells is mediated by the chemokine receptor CC-CKR-5 Nature 1996 381 667 673 10.1038/381667a0 8649512

[B49] Lapham CK Ouyang J Chandrasekhar B Nguyen NY Dimitrov DS Golding H Evidence for cell-surface association between fusin and the CD4-gp120 complex in human cell lines Science 1996 274 602 605 10.1126/science.274.5287.602 8849450

[B50] Wu L Gerard NP Wyatt R Choe H Parolin C Ruffing N Borsetti A Cardoso AA Desjardin E Newman W CD4-induced interaction of primary HIV-1 gp120 glycoproteins with the chemokine receptor CCR-5 Nature 1996 384 179 183 10.1038/384179a0 8906795

[B51] Trkola A Dragic T Arthos J Binley JM Olson WC Allaway GP Cheng-Mayer C Robinson J Maddon PJ Moore JP CD4-dependent, antibody-sensitive interactions between HIV-1 and its co-receptor CCR-5 Nature 1996 384 184 187 10.1038/384184a0 8906796

[B52] Hill CM Deng H Unutmaz D Kewalramani VN Bastiani L Gorny MK Zolla-Pazner S Littman DR Envelope glycoproteins from human immunodeficiency virus types 1 and 2 and simian immunodeficiency virus can use human CCR5 as a coreceptor for viral entry and make direct CD4-dependent interactions with this chemokine receptor J Virol 1997 71 6296 6304 9261346 10.1128/jvi.71.9.6296-6304.1997 PMC191902

[B53] Ugolini S Moulard M Mondor I Barois N Demandolx D Hoxie J Brelot A Alizon M Davoust J Sattentau QJ HIV-1 gp120 induces an association between CD4 and the chemokine receptor CXCR4 J Immunol 1997 159 3000 3008 9300725

[B54] Iyengar S Hildreth JE Schwartz DH Actin-dependent receptor colocalization required for human immunodeficiency virus entry into host cells J Virol 1998 72 5251 5255 9573299 10.1128/jvi.72.6.5251-5255.1998 PMC110111

[B55] Jimenez-Baranda S Gomez-Mouton C Rojas A Martinez-Prats L Mira E Ana Lacalle R Valencia A Dimitrov DS Viola A Delgado R Filamin-A regulates actin-dependent clustering of HIV receptors Nat Cell Biol 2007 9 838 846 10.1038/ncb1610 17572668

[B56] Barrero-Villar M Cabrero JR Gordon-Alonso M Barroso-Gonzalez J Alvarez-Losada S Munoz-Fernandez MA Sanchez-Madrid F Valenzuela-Fernandez A Moesin is required for HIV-1-induced CD4-CXCR4 interaction, F-actin redistribution, membrane fusion and viral infection in lymphocytes J Cell Sci 2009 122 103 113 10.1242/jcs.035873 19066282

[B57] Pontow SE Heyden NV Wei S Ratner L Actin cytoskeletal reorganizations and coreceptor-mediated activation of rac during human immunodeficiency virus-induced cell fusion Journal of Virology 2004 78 7138 7147 10.1128/JVI.78.13.7138-7147.2004 15194790 PMC421652

[B58] Harmon B Ratner L Induction of the Galpha(q) signaling cascade by the human immunodeficiency virus envelope is required for virus entry J Virol 2008 82 9191 9205 10.1128/JVI.00424-08 18632858 PMC2546909

[B59] Harmon B Campbell N Ratner L Role of Abl kinase and the Wave2 signaling complex in HIV-1 entry at a post-hemifusion step PLoS Pathog 2010 6 e1000956 10.1371/journal.ppat.1000956 20585556 PMC2887473

[B60] Lapham CK Zaitseva MB Lee S Romanstseva T Golding H Fusion of monocytes and macrophages with HIV-1 correlates with biochemical properties of CXCR4 and CCR5 Nat Med 1999 5 303 308 10.1038/6523 10086386

[B61] Xiao X Wu L Stantchev TS Feng YR Ugolini S Chen H Shen Z Riley JL Broder CC Sattentau QJ Dimitrov DS Constitutive cell surface association between CD4 and CCR5 Proc Natl Acad Sci U S A 1999 96 7496 7501 10.1073/pnas.96.13.7496 10377443 PMC22114

[B62] Singer II Scott S Kawka DW Chin J Daugherty BL DeMartino JA DiSalvo J Gould SL Lineberger JE Malkowitz L CCR5, CXCR4, and CD4 are clustered and closely apposed on microvilli of human macrophages and T cells J Virol 2001 75 3779 3790 10.1128/JVI.75.8.3779-3790.2001 11264367 PMC114869

[B63] Baker AM Sauliere A Gaibelet G Lagane B Mazeres S Fourage M Bachelerie F Salome L Lopez A Dumas F CD4 interacts constitutively with multiple CCR5 at the plasma membrane of living cells. A fluorescence recovery after photobleaching at variable radii approach J Biol Chem 2007 282 35163 35168 10.1074/jbc.M705617200 17855336

[B64] Toth PT Ren D Miller RJ Regulation of CXCR4 receptor dimerization by the chemokine SDF-1alpha and the HIV-1 coat protein gp120: a fluorescence resonance energy transfer (FRET) study J Pharmacol Exp Ther 2004 310 8 17 10.1124/jpet.103.064956 15014135

[B65] Seror C Melki MT Subra F Raza SQ Bras M Saidi H Nardacci R Voisin L Paoletti A Law F Extracellular ATP acts on P2Y2 purinergic receptors to facilitate HIV-1 infection J Exp Med 2011 208 1823 1834 10.1084/jem.20101805 21859844 PMC3171090

[B66] Platt EJ Wehrly K Kuhmann SE Chesebro B Kabat D Effects of CCR5 and CD4 cell surface concentrations on infections by macrophagetropic isolates of human immunodeficiency virus type 1 J Virol 1998 72 2855 2864 9525605 10.1128/jvi.72.4.2855-2864.1998 PMC109730

[B67] Layne SP Merges MJ Dembo M Spouge JL Nara PL HIV requires multiple gp120 molecules for CD4-mediated infection Nature 1990 346 277 279 10.1038/346277a0 2374593

[B68] Kuhmann SE Platt EJ Kozak SL Kabat D Cooperation of multiple CCR5 coreceptors is required for infections by human immunodeficiency virus type 1 J Virol 2000 74 7005 7015 10.1128/JVI.74.15.7005-7015.2000 10888639 PMC112217

[B69] Sougrat R Bartesaghi A Lifson JD Bennett AE Bess JW Zabransky DJ Subramaniam S Electron tomography of the contact between T cells and SIV/HIV-1: implications for viral entry PLoS Pathog 2007 3 e63 10.1371/journal.ppat.0030063 17480119 PMC1864992

[B70] Del Real G Jimenez-Baranda S Lacalle RA Mira E Lucas P Gomez-Mouton C Carrera AC Martinez AC Manes S Blocking of HIV-1 infection by targeting CD4 to nonraft membrane domains J Exp Med 2002 196 293 301 10.1084/jem.20020308 12163558 PMC2193941

[B71] Manes S del Real G Lacalle RA Lucas P Gomez-Mouton C Sanchez-Palomino S Delgado R Alcami J Mira E Martinez AC Membrane raft microdomains mediate lateral assemblies required for HIV-1 infection EMBO Rep 2000 1 190 196 10.1093/embo-reports/kvd025 11265761 PMC1084257

[B72] Popik W Alce TM Au WC Human immunodeficiency virus type 1 uses lipid raft-colocalized CD4 and chemokine receptors for productive entry into CD4(+) T cells J Virol 2002 76 4709 4722 10.1128/JVI.76.10.4709-4722.2002 11967288 PMC136131

[B73] Kamiyama H Yoshii H Tanaka Y Sato H Yamamoto N Kubo Y Raft localization of CXCR4 is primarily required for X4-tropic human immunodeficiency virus type 1 infection Virology 2009 386 23 31 10.1016/j.virol.2008.12.033 19178925

[B74] Nguyen DH Giri B Collins G Taub DD Dynamic reorganization of chemokine receptors, cholesterol, lipid rafts, and adhesion molecules to sites of CD4 engagement Exp Cell Res 2005 304 559 569 10.1016/j.yexcr.2004.11.022 15748900

[B75] Popik W Alce TM CD4 receptor localized to non-raft membrane microdomains supports HIV-1 entry. Identification of a novel raft localization marker in CD4 J Biol Chem 2004 279 704 712 14570906 10.1074/jbc.M306380200

[B76] Percherancier Y Lagane B Planchenault T Staropoli I Altmeyer R Virelizier JL Arenzana-Seisdedos F Hoessli DC Bachelerie F HIV-1 entry into T-cells is not dependent on CD4 and CCR5 localization to sphingolipid-enriched, detergent-resistant, raft membrane domains J Biol Chem 2003 278 3153 3161 10.1074/jbc.M207371200 12431990

[B77] Balabanian K Harriague J Decrion C Lagane B Shorte S Baleux F Virelizier JL Arenzana-Seisdedos F Chakrabarti LA CXCR4-tropic HIV-1 envelope glycoprotein functions as a viral chemokine in unstimulated primary CD4+ T lymphocytes J Immunol 2004 173 7150 7160 15585836 10.4049/jimmunol.173.12.7150

[B78] Chang MI Panorchan P Dobrowsky TM Tseng Y Wirtz D Single-molecule analysis of human immunodeficiency virus type 1 gp120-receptor interactions in living cells J Virol 2005 79 14748 14755 10.1128/JVI.79.23.14748-14755.2005 16282475 PMC1287567

[B79] Gallo SA Reeves JD Garg H Foley B Doms RW Blumenthal R Kinetic studies of HIV-1 and HIV-2 envelope glycoprotein-mediated fusion Retrovirology 2006 3 90 10.1186/1742-4690-3-90 17144914 PMC1693918

[B80] Gallo SA Puri A Blumenthal R HIV-1 gp41 six-helix bundle formation occurs rapidly after the engagement of gp120 by CXCR4 in the HIV-1 Env-mediated fusion process Biochemistry 2001 40 12231 12236 10.1021/bi0155596 11591141

[B81] Readinger JA Schiralli GM Jiang JK Thomas CJ August A Henderson AJ Schwartzberg PL Selective targeting of ITK blocks multiple steps of HIV replication Proc Natl Acad Sci U S A 2008 105 6684 6689 10.1073/pnas.0709659105 18443296 PMC2365562

[B82] Weissman D Rabin RL Arthos J Rubbert A Dybul M Swofford R Venkatesan S Farber JM Fauci AS Macrophage-tropic HIV and SIV envelope proteins induce a signal through the CCR5 chemokine receptor Nature 1997 389 981 985 10.1038/40173 9353123

[B83] Komano J Miyauchi K Matsuda Z Yamamoto N Inhibiting the Arp2/3 complex limits infection of both intracellular mature vaccinia virus and primate lentiviruses Mol Biol Cell 2004 15 5197 5207 10.1091/mbc.E04-04-0279 15385624 PMC532003

[B84] Naghavi MH Valente S Hatziioannou T de Los Santos K Wen Y Mott C Gundersen GG Goff SP Moesin regulates stable microtubule formation and limits retroviral infection in cultured cells Embo J 2007 26 41 52 10.1038/sj.emboj.7601475 17170707 PMC1782362

[B85] Arthur LO Bess JW Sowder RC Benveniste RE Mann DL Chermann JC Henderson LE Cellular proteins bound to immunodeficiency viruses: implications for pathogenesis and vaccines Science 1992 258 1935 1938 10.1126/science.1470916 1470916

[B86] Ott DE Coren LV Johnson DG Kane BP Sowder RC Kim YD Fisher RJ Zhou XZ Lu KP Henderson LE Actin-binding cellular proteins inside human immunodeficiency virus type 1 Virology 2000 266 42 51 10.1006/viro.1999.0075 10612659

[B87] Ott DE Coren LV Kane BP Busch LK Johnson DG Sowder RC Chertova EN Arthur LO Henderson LE Cytoskeletal proteins inside human immunodeficiency virus type 1 virions J Virol 1996 70 7734 7743 8892894 10.1128/jvi.70.11.7734-7743.1996 PMC190843

[B88] Junghans RP Duesberg PH Knight CA In vitro synthesis of full-length DNA transcripts of Rous sarcoma virus RNA by viral DNA polymerase Proc Natl Acad Sci U S A 1975 72 4895 4899 10.1073/pnas.72.12.4895 174081 PMC388839

[B89] Zhang H Zhang Y Spicer TP Abbott LZ Abbott M Poiesz BJ Reverse transcription takes place within extracellular HIV-1 virions: potential biological significance AIDS Res Hum Retroviruses 1993 9 1287 1296 10.1089/aid.1993.9.1287 8142146

[B90] Lori F di Marzo Veronese F de Vico AL Lusso P Reitz MS Gallo RC Viral DNA carried by human immunodeficiency virus type 1 virions J Virol 1992 66 5067 5074 1378514 10.1128/jvi.66.8.5067-5074.1992 PMC241368

[B91] Trono D Partial reverse transcripts in virions from human immunodeficiency and murine leukemia viruses J Virol 1992 66 4893 4900 1378513 10.1128/jvi.66.8.4893-4900.1992 PMC241327

[B92] Warrilow D Stenzel D Harrich D Isolated HIV-1 core is active for reverse transcription Retrovirology 2007 4 77 10.1186/1742-4690-4-77 17956635 PMC2169257

[B93] Naldini L Blomer U Gallay P Ory D Mulligan R Gage FH Verma IM Trono D In vivo gene delivery and stable transduction of nondividing cells by a lentiviral vector Science 1996 272 263 267 10.1126/science.272.5259.263 8602510

[B94] Reiser J Harmison G Kluepfel-Stahl S Brady RO Karlsson S Schubert M Transduction of nondividing cells using pseudotyped defective high-titer HIV type 1 particles Proc Natl Acad Sci U S A 1996 93 15266 15271 10.1073/pnas.93.26.15266 8986799 PMC26392

[B95] Varmus HE Padgett T Heasley S Simon G Bishop JM Cellular functions are required for the synthesis and integration of avian sarcoma virus-specific DNA Cell 1977 11 307 319 10.1016/0092-8674(77)90047-2 196759

[B96] Zhang H Bagasra O Niikura M Poiesz BJ Pomerantz RJ Intravirion reverse transcripts in the peripheral blood plasma on human immunodeficiency virus type 1-infected individuals J Virol 1994 68 7591 7597 7933148 10.1128/jvi.68.11.7591-7597.1994 PMC237208

[B97] Zhang H Zhang Y Spicer T Henrard D Poiesz BJ Nascent human immunodeficiency virus type 1 reverse transcription occurs within an enveloped particle J Virol 1995 69 3675 3682 7745716 10.1128/jvi.69.6.3675-3682.1995 PMC189083

[B98] Zhang H Dornadula G Orenstein J Pomerantz RJ Morphologic changes in human immunodeficiency virus type 1 virions secondary to intravirion reverse transcription: evidence indicating that reverse transcription may not take place within the intact viral core J Hum Virol 2000 3 165 172 10881997

[B99] Forshey BM von Schwedler U Sundquist WI Aiken C Formation of a human immunodeficiency virus type 1 core of optimal stability is crucial for viral replication J Virol 2002 76 5667 5677 10.1128/JVI.76.11.5667-5677.2002 11991995 PMC137032

[B100] Dismuke DJ Aiken C Evidence for a functional link between uncoating of the human immunodeficiency virus type 1 core and nuclear import of the viral preintegration complex J Virol 2006 80 3712 3720 10.1128/JVI.80.8.3712-3720.2006 16571788 PMC1440469

[B101] Warrilow D Harrich D HIV-1 replication from after cell entry to the nuclear periphery Curr HIV Res 2007 5 293 299 10.2174/157016207780636579 17504171

[B102] Merrifield CJ Perrais D Zenisek D Coupling between clathrin-coated-pit invagination, cortactin recruitment, and membrane scission observed in live cells Cell 2005 121 593 606 10.1016/j.cell.2005.03.015 15907472

[B103] Brun S Solignat M Gay B Bernard E Chaloin L Fenard D Devaux C Chazal N Briant L VSV-G pseudotyping rescues HIV-1 CA mutations that impair core assembly or stability Retrovirology 2008 5 57 10.1186/1742-4690-5-57 18605989 PMC2474847

[B104] Arhel N Revisiting HIV-1 uncoating Retrovirology 2011 7 96 21083892 10.1186/1742-4690-7-96 PMC2998454

[B105] Auewarakul P Wacharapornin P Srichatrapimuk S Chutipongtanate S Puthavathana P Uncoating of HIV-1 requires cellular activation Virology 2005 337 93 101 10.1016/j.virol.2005.02.028 15882886

[B106] Marcy Y Prost J Carlier MF Sykes C Forces generated during actin-based propulsion: a direct measurement by micromanipulation Proc Natl Acad Sci U S A 2004 101 5992 5997 10.1073/pnas.0307704101 15079054 PMC395911

[B107] Abraham VC Krishnamurthi V Taylor DL Lanni F The actin-based nanomachine at the leading edge of migrating cells Biophys J 1999 77 1721 1732 10.1016/S0006-3495(99)77018-9 10465781 PMC1300458

[B108] Bukrinskaya A Brichacek B Mann A Stevenson M Establishment of a functional human immunodeficiency virus type 1 (HIV-1) reverse transcription complex involves the cytoskeleton J Exp Med 1998 188 2113 2125 10.1084/jem.188.11.2113 9841925 PMC2212381

[B109] Wu Y Beddall MH Marsh JW Rev-dependent lentiviral expression vector Retrovirology 2007 4 12 10.1186/1742-4690-4-12 17286866 PMC1797186

[B110] Wu Y Beddall MH Marsh JW Rev-dependent indicator T cell line Current HIV Research 2007 5 395 403 10.2174/157016207781024018 PMC2366165 17627502

[B111] Kestler HWd Ringler DJ Mori K Panicali DL Sehgal PK Daniel MD Desrosiers RC Importance of the nef gene for maintenance of high virus loads and for development of AIDS Cell 1991 65 651 662 10.1016/0092-8674(91)90097-I 2032289

[B112] Chowers MY Spina CA Kwoh TJ Fitch NJ Richman DD Guatelli JC Optimal infectivity in vitro of human immunodeficiency virus type 1 requires an intact nef gene J Virol 1994 68 2906 2914 8151761 10.1128/jvi.68.5.2906-2914.1994 PMC236779

[B113] Miller MD Warmerdam MT Gaston I Greene WC Feinberg MB The human immunodeficiency virus-1 nef gene product: a positive factor for viral infection and replication in primary lymphocytes and macrophages J Exp Med 1994 179 101 113 10.1084/jem.179.1.101 8270859 PMC2191317

[B114] Aiken C Trono D Nef stimulates human immunodeficiency virus type 1 proviral DNA synthesis J Virol 1995 69 5048 5056 7541845 10.1128/jvi.69.8.5048-5056.1995 PMC189322

[B115] Chowers MY Pandori MW Spina CA Richman DD Guatelli JC The growth advantage conferred by HIV-1 nef is determined at the level of viral DNA formation and is independent of CD4 downregulation Virology 1995 212 451 457 10.1006/viro.1995.1502 7571414

[B116] Schwartz O Marechal V Danos O Heard JM Human immunodeficiency virus type 1 Nef increases the efficiency of reverse transcription in the infected cell J Virol 1995 69 4053 4059 7539505 10.1128/jvi.69.7.4053-4059.1995 PMC189139

[B117] Kotov A Zhou J Flicker P Aiken C Association of Nef with the human immunodeficiency virus type 1 core J Virol 1999 73 8824 8830 10482638 10.1128/jvi.73.10.8824-8830.1999 PMC112905

[B118] Forshey BM Aiken C Disassembly of human immunodeficiency virus type 1 cores in vitro reveals association of Nef with the subviral ribonucleoprotein complex J Virol 2003 77 4409 4414 10.1128/JVI.77.7.4409-4414.2003 12634398 PMC150647

[B119] Zhou Y Zhang H Siliciano JD Siliciano RF Kinetics of human immunodeficiency virus type 1 decay following entry into resting CD4+ T cells J Virol 2005 79 2199 2210 10.1128/JVI.79.4.2199-2210.2005 15681422 PMC546571

[B120] Zack JA Arrigo SJ Weitsman SR Go AS Haislip A Chen IS HIV-1 entry into quiescent primary lymphocytes: molecular analysis reveals a labile, latent viral structure Cell 1990 61 213 222 10.1016/0092-8674(90)90802-L 2331748

[B121] Korin YD Zack JA Nonproductive human immunodeficiency virus type 1 infection in nucleoside-treated G0 lymphocytes J Virol 1999 73 6526 6532 10400748 10.1128/jvi.73.8.6526-6532.1999 PMC112735

[B122] Campbell GR Spector SA CCL2 increases X4-tropic HIV-1 entry into resting CD4+ T cells J Biol Chem 2008 283 30745 30753 10.1074/jbc.M804112200 18784079 PMC2576528

[B123] Miller MD Farnet CM Bushman FD Human immunodeficiency virus type 1 preintegration complexes: studies of organization and composition J Virol 1997 71 5382 5390 9188609 10.1128/jvi.71.7.5382-5390.1997 PMC191777

[B124] McDonald D Vodicka MA Lucero G Svitkina TM Borisy GG Emerman M Hope TJ Visualization of the intracellular behavior of HIV in living cells J Cell Biol 2002 159 441 452 10.1083/jcb.200203150 12417576 PMC2173076

[B125] Arhel N Genovesio A Kim KA Miko S Perret E Olivo-Marin JC Shorte S Charneau P Quantitative four-dimensional tracking of cytoplasmic and nuclear HIV-1 complexes Nat Methods 2006 3 817 824 10.1038/nmeth928 16990814

[B126] Kimpton J Emerman M Detection of replication-competent and pseudotyped human immunodeficiency virus with a sensitive cell line on the basis of activation of an integrated beta-galactosidase gene J Virol 1992 66 2232 2239 1548759 10.1128/jvi.66.4.2232-2239.1992 PMC289016

[B127] Fredericksen BL Wei BL Yao J Luo T Garcia JV Inhibition of endosomal/lysosomal degradation increases the infectivity of human immunodeficiency virus J Virol 2002 76 11440 11446 10.1128/JVI.76.22.11440-11446.2002 12388705 PMC136743

[B128] Wei BL Denton PW O'Neill E Luo T Foster JL Garcia JV Inhibition of lysosome and proteasome function enhances human immunodeficiency virus type 1 infection J Virol 2005 79 5705 5712 10.1128/JVI.79.9.5705-5712.2005 15827185 PMC1082736

[B129] Zamborlini A Lehmann-Che J Clave E Giron ML Tobaly-Tapiero J Roingeard P Emiliani S Toubert A de The H Saib A Centrosomal pre-integration latency of HIV-1 in quiescent cells Retrovirology 2007 4 63 10.1186/1742-4690-4-63 17845727 PMC2014762

[B130] Suzuki Y Craigie R The road to chromatin - nuclear entry of retroviruses Nat Rev Microbiol 2007 5 187 196 10.1038/nrmicro1579 17304248

[B131] Yoder A Guo J Yu D Cui Z Zhang XE Wu Y Effects of Microtubule Modulators on HIV-1 Infection of Transformed and Resting CD4 T Cells J Virol 2011 85 3020 3024 10.1128/JVI.02462-10 21209111 PMC3067922

[B132] Saleh S Solomon A Wightman F Xhilaga M Cameron PU Lewin SR CCR7 ligands CCL19 and CCL21 increase permissiveness of resting memory CD4+ T cells to HIV-1 infection: a novel model of HIV-1 latency Blood 2007 110 4161 4164 10.1182/blood-2007-06-097907 17881634

[B133] Cameron PU Saleh SM Sallman G Solomon A Wightman F Mark J Hones K Anderson J Jaworowski A Lewin SR Latent HIV infection can be established in resting CD4+ T-cells in vitro following incubation with multiple diverse chemokines which facilitate nuclear import of the pre-integration complex4th international workship in HIV persistence during therapy 2009 2009 8 11

[B134] Gey GO Bang FB Gey MK Responses of a variety of normal and malignant cells to continuous cultivation, and some practical applications of these responses to problems in the biology of disease Ann N Y Acad Sci 1954 58 976 999 10.1111/j.1749-6632.1954.tb45886.x 14350495

[B135] Bunnell BA Muul LM Donahue RE Blaese RM Morgan RA High-efficiency retroviral-mediated gene transfer into human and nonhuman primate peripheral blood lymphocytes Proc Natl Acad Sci U S A 1995 92 7739 7743 10.1073/pnas.92.17.7739 7644487 PMC41221

[B136] Forestell SP Dando JS Bohnlein E Rigg RJ Improved detection of replication-competent retrovirus J Virol Methods 1996 60 171 178 10.1016/0166-0934(96)02052-6 8844623

[B137] Ho WZ Cherukuri R Ge SD Cutilli JR Song L Whitko S Douglas SD Centrifugal enhancement of human immunodeficiency virus type 1 infection and human cytomegalovirus gene expression in human primary monocyte/macrophages in vitro J Leukoc Biol 1993 53 208 212 7680371 10.1002/jlb.53.2.208

[B138] O'Doherty U Swiggard WJ Malim MH Human immunodeficiency virus type 1 spinoculation enhances infection through virus binding J Virol 2000 74 10074 10080 10.1128/JVI.74.21.10074-10080.2000 11024136 PMC102046

[B139] Cocchi F DeVico AL Garzino-Demo A Cara A Gallo RC Lusso P The V3 domain of the HIV-1 gp120 envelope glycoprotein is critical for chemokine-mediated blockade of infection Nat Med 1996 2 1244 1247 10.1038/nm1196-1244 8898753

[B140] Farzan M Choe H Martin KA Sun Y Sidelko M Mackay CR Gerard NP Sodroski J Gerard C HIV-1 entry and macrophage inflammatory protein-1beta-mediated signaling are independent functions of the chemokine receptor CCR5 Journal of Biological Chemistry 1997 272 6854 6857 10.1074/jbc.272.11.6854 9054370

[B141] Alkhatib G Locati M Kennedy PE Murphy PM Berger EA HIV-1 coreceptor activity of CCR5 and its inhibition by chemokines: independence from G protein signaling and importance of coreceptor downmodulation Virology 1997 234 340 348 10.1006/viro.1997.8673 9268166

[B142] Gosling J Monteclaro FS Atchison RE Arai H Tsou CL Goldsmith MA Charo IF Molecular uncoupling of C-C chemokine receptor 5-induced chemotaxis and signal transduction from HIV-1 coreceptor activity Proc Natl Acad Sci U S A 1997 94 5061 5066 10.1073/pnas.94.10.5061 9144190 PMC24631

[B143] Aramori I Ferguson SS Bieniasz PD Zhang J Cullen B Cullen MG Molecular mechanism of desensitization of the chemokine receptor CCR-5: receptor signaling and internalization are dissociable from its role as an HIV-1 co-receptor Embo J 1997 16 4606 4616 10.1093/emboj/16.15.4606 9303305 PMC1170087

[B144] Amara A Gall SL Schwartz O Salamero J Montes M Loetscher P Baggiolini M Virelizier JL Arenzana-Seisdedos F HIV coreceptor downregulation as antiviral principle: SDF-1alpha-dependent internalization of the chemokine receptor CXCR4 contributes to inhibition of HIV replication J Exp Med 1997 186 139 146 10.1084/jem.186.1.139 9207008 PMC2198965

[B145] Lu Z Berson JF Chen Y Turner JD Zhang T Sharron M Jenks MH Wang Z Kim J Rucker J Evolution of HIV-1 coreceptor usage through interactions with distinct CCR5 and CXCR4 domains Proc Natl Acad Sci U S A 1997 94 6426 6431 10.1073/pnas.94.12.6426 9177234 PMC21066

[B146] Doranz BJ Orsini MJ Turner JD Hoffman TL Berson JF Hoxie JA Peiper SC Brass LF Doms RW Identification of CXCR4 domains that support coreceptor and chemokine receptor functions J Virol 1999 73 2752 2761 10074122 10.1128/jvi.73.4.2752-2761.1999 PMC104032

[B147] Brelot A Heveker N Montes M Alizon M Identification of residues of CXCR4 critical for human immunodeficiency virus coreceptor and chemokine receptor activities J Biol Chem 2000 275 23736 23744 10.1074/jbc.M000776200 10825158

[B148] Amara A Vidy A Boulla G Mollier K Garcia-Perez J Alcami J Blanpain C Parmentier M Virelizier JL Charneau P Arenzana-Seisdedos F G protein-dependent CCR5 signaling is not required for efficient infection of primary T lymphocytes and macrophages by R5 human immunodeficiency virus type 1 isolates Journal of Virology 2003 77 2550 2558 10.1128/JVI.77.4.2550-2558.2003 12551993 PMC141084

[B149] Stolp B Fackler OT How HIV takes advantage of the cytoskeleton in entry and replication Viruses 2011 3 293 311 10.3390/v3040293 21994733 PMC3185699

[B150] Taylor MP Koyuncu OO Enquist LW Subversion of the actin cytoskeleton during viral infection Nat Rev Microbiol 2011 9 427 439 10.1038/nrmicro2574 21522191 PMC3229036

[B151] Lehmann M Nikolic DS Piguet V How HIV-1 takes advantage of the cytoskeleton during replication and cell-to-cell transmission Viruses 2011 3 1757 1776 10.3390/v3091757 21994805 PMC3187690

[B152] Jolly C Kashefi K Hollinshead M Sattentau QJ HIV-1 cell to cell transfer across an Env-induced, actin-dependent synapse J Exp Med 2004 199 283 293 10.1084/jem.20030648 14734528 PMC2211771

[B153] Vasiliver-Shamis G Cho MW Hioe CE Dustin ML Human immunodeficiency virus type 1 envelope gp120-induced partial T-cell receptor signaling creates an F-actin-depleted zone in the virological synapse J Virol 2009 83 11341 11355 10.1128/JVI.01440-09 19710135 PMC2772796

[B154] Jolly C Mitar I Sattentau QJ Requirement for an intact T-cell actin and tubulin cytoskeleton for efficient assembly and spread of human immunodeficiency virus type 1 J Virol 2007 81 5547 5560 10.1128/JVI.01469-06 17360745 PMC1900271

[B155] Gladnikoff M Shimoni E Gov NS Rousso I Retroviral assembly and budding occur through an actin-driven mechanism Biophys J 2009 97 2419 2428 10.1016/j.bpj.2009.08.016 19883584 PMC2770610

[B156] Cooper J Liu L Woodruff EA Taylor HE Goodwin JS D'Aquila RT Spearman P Hildreth JE Dong X Filamin A protein interacts with human immunodeficiency virus type 1 Gag protein and contributes to productive particle assembly J Biol Chem 2011 286 28498 28510 10.1074/jbc.M111.239053 21705339 PMC3151092

[B157] Graziano F Elia C Laudanna C Poli G Alfano M Urokinase plasminogen activator inhibits HIV virion release from macrophage-differentiated chronically infected cells via activation of RhoA and PKCepsilon PLoS One 2011 6 e23674 10.1371/journal.pone.0023674 21858203 PMC3157461

[B158] Ansari AW Bhatnagar N Dittrich-Breiholz O Kracht M Schmidt RE Heiken H Host chemokine (C-C motif) ligand-2 (CCL2) is differentially regulated in HIV type 1 (HIV-1)-infected individuals Int Immunol 2006 18 1443 1451 10.1093/intimm/dxl078 16916890 PMC7108614

[B159] Damas JK Landro L Fevang B Heggelund L Froland SS Aukrust P Enhanced levels of the CCR7 ligands CCL19 and CCL21 in HIV infection: correlation with viral load, disease progression and response to highly active antiretroviral therapy Aids 2009 23 135 138 10.1097/QAD.0b013e32831cf595 19050397

[B160] Damas JK Landro L Fevang B Heggelund L Tjonnfjord GE Floisand Y Halvorsen B Froland SS Aukrust P Homeostatic chemokines CCL19 and CCL21 promote inflammation in human immunodeficiency virus-infected patients with ongoing viral replication Clin Exp Immunol 2009 157 400 407 10.1111/j.1365-2249.2009.03976.x 19664149 PMC2745035

[B161] Wu Y Yoder A Yu D Wang W Liu J Barrett T Wheeler D Schlauch K Cofilin activation in peripheral CD4 T cells of HIV-1 infected patients: a pilot study Retrovirology 2008 5 95 10.1186/1742-4690-5-95 18928553 PMC2576353

[B162] Howard AD McAllister G Feighner SD Liu Q Nargund RP Van der Ploeg LH Patchett AA Orphan G-protein-coupled receptors and natural ligand discovery Trends Pharmacol Sci 2001 22 132 140 11239576 10.1016/s0165-6147(00)01636-9

